# Inverse scattering transform analysis of rogue waves using local periodization procedure

**DOI:** 10.1038/srep29238

**Published:** 2016-07-07

**Authors:** Stéphane Randoux, Pierre Suret, Gennady El

**Affiliations:** 1 Univ. Lille, CNRS, UMR 8523 - PhLAM - Physique des Lasers Atomes et Molécules, F-59000 Lille, France; 2 Department of Mathematical Sciences, Loughborough University, Loughborough LE11 3TU, United Kingdom

## Abstract

The nonlinear Schrödinger equation (NLSE) stands out as the dispersive nonlinear partial differential equation that plays a prominent role in the modeling and understanding of the wave phenomena relevant to many fields of nonlinear physics. The question of random input problems in the one-dimensional and integrable NLSE enters within the framework of integrable turbulence, and the specific question of the formation of rogue waves (RWs) has been recently extensively studied in this context. The determination of exact analytic solutions of the focusing 1D-NLSE prototyping RW events of statistical relevance is now considered as the problem of central importance. Here we address this question from the perspective of the inverse scattering transform (IST) method that relies on the integrable nature of the wave equation. We develop a conceptually new approach to the RW classification in which appropriate, locally coherent structures are specifically isolated from a globally incoherent wave train to be subsequently analyzed by implementing a numerical IST procedure relying on a spatial periodization of the object under consideration. Using this approach we extend the existing classifications of the prototypes of RWs from standard breathers and their collisions to more general nonlinear modes characterized by their nonlinear spectra.

There is currently much research interest in the subject of the formation of rogue waves (RWs). The traditional notion of RWs is related to rare events of large amplitude that appear unpredictably on the ocean surface^1^,^2^. From the optical fiber experiment performed by Solli *et al*. in ref. 3, it has been understood that RWs are ubiquitous phenomena observable not only in oceanography but also in many other physical contexts^4^. Although the unique mechanism of the RW formation cannot be drawn^4^,^5^,^6^,^7^, it is now understood that the one-dimensional focusing nonlinear Schrödinger equation (1D-NLSE) provides a universal description of a variety of nonlinear localization effects that are compatible with RW events^6^,^7^,^8^. The best known analytical models for RWs are solitons on finite background (SFBs) which represent exact homoclinic solutions of the 1D-NLSE having the far-field behavior of a finite-amplitude plane wave and at the same time exhibiting local peak amplitudes compatible with the threshold definition of rogue events^6^,^8^,^9^,^10^,^11^. Taking specific and carefully designed initial conditions, many SFBs have now been observed in well-controlled experiments performed in several physical systems^12^,^13^,^14^,^15^,^16^,^17^,^18^,^19^.

Taking random initial conditions in wave problems ruled by the 1D-NLSE is very pertinent to the study of RWs because the randomness of the initial condition opens the way for the statistical treatment inherent in any physically realistic RW description^6^,^20^,^21^. The theoretical analysis of random input problems in integrable equations such as the 1D-NLSE enters within the framework of integrable turbulence^21^,^22^,^23^,^24^,^25^. Regarding the focusing 1D-NLSE, it has been recently shown that the statistics of the field that is measured at long evolution time strongly depends on the statistics of the random initial condition. A plane wave perturbed with a random small noise has been found to produce a field eventually assuming the gaussian statistics^23^. On the other hand, heavy-tailed deviations from gaussianity have been observed for random fields having gaussian statistics at the initial stage^21^,^26^. The important questions related to the relationship between the initial condition and the formation of RWs have been recently investigated in the framework of the inverse scattering transform (IST) method^27^. It has been also shown that random initial conditions can excite a range of SFBs well described by exact analytic solutions of the 1D-NLSE^6^,^8^,^9^,^20^,^21^,^26^ and that 1D-NLSE RWs can arise from collisions between some solitons^8^,^18^,^20^.

The question of the identification and classification of NLSE RWs is a current issue of importance. So far, this question has been mainly investigated by using numerical simulations^6^,^8^,^9^,^20^,^21^. However the first real-time and direct observation of RWs generated from the propagation of partially coherent waves in optical fibers has been recently reported in ref. 26. In these experiments based on the time lens technique, breather-like structures such as Peregrine solitons (PSs) have been shown to emerge locally from the random background. In the majority of the approaches reported so far, RW objects are first specifically isolated from random wave trains, and their classification relies on fitting procedures in which the profiles of interest are locally compared with well-known analytic SFB solutions of the focusing 1D-NLSE^6^,^8^,^9^,^20^,^21^,^26^. In this paper, we propose a conceptually new approach to the characterization of RWs that are observed in wave systems ruled by the focusing 1D-NLSE. We introduce a method relying on the integrable nature of the 1D-NLSE to compute spectral portraits of localized structures by using the direct scattering transform, which forms an integral part of the IST method. Our approach extends the existing rigid identification of RWs with one of the prototypical exact breather solutions such as Akhmediev breathers (ABs), Kuznetsov-Ma (KM) solitons, PSs or the higher-order SFB solutions describing their collisions. Instead, we show that RWs observed in random NLSE solutions represent more general wave forms which may or may not be very close to one of the prototypical SFBs.

The IST is a well-established method for solving nonlinear integrable partial differential equations. It has been shown recently that the IST method can provide a new approach to overcome transmission limitations in fiber communication channels by encoding information in the nonlinear IST spectrum^28^,^29^. Here, we exploit the fact that the IST can be used to determine spectral portraits of localized structures found in some wave trains of interest. These spectral portraits (for convenience we shall also call them the *IST spectra*) provide very accurate signatures of the localized structures and they can be compared to the spectral signatures of fundamental solitons and SFBs, which are well-known from the IST theory. Note that the IST has already been introduced as a tool for nonlinear Fourier analysis of random wave trains^30^,^31^,^32^,^33^. This tool has been successfully implemented in several circumstances to determine the content of random wave trains in terms of nonlinear oscillating modes. In particular, the IST analysis has been used to analyze the soliton content in freak (rogue) wave time series^34^ and more recently, to evidence the presence of soliton turbulence in shallow water ocean surface waves^35^. Regarding the specific question of the prediction of RWs, previous numerical computations of IST spectra have shown that the development of RWs can be statistically correlated with the proximity to homoclinic solutions of the 1D-NLSE^36^. Recently the process of RW formation has been studied by computing *global* IST spectra characterizing multiple random fluctuations found inside a box having a large size^27^. Here, we develop a new *local* approach in which the objects compatible with prototypes of RWs are specifically isolated from a wave train to be subsequently analyzed using a numerical IST procedure that relies on a spatial periodization of the object under consideration. With this conceptually new approach, we determine the most essential nonlinear modes composing the RW under consideration and expand the existing paradigm that observable RWs are necessarily described by the standard SFB analytic solutions of the focusing 1D-NLSE.

## Inverse Scattering Transform method to compute spectral portraits

### Spectral portraits of some soliton solutions of the 1D-NLSE

We consider the focusing 1D-NLSE in the form


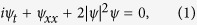


where *ψ*(*x*, *t*) is a complex wave envelope changing in space *x* and time *t*. In the IST method, the NLSE is represented as the compatibility condition of two linear equations^37^,


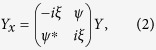



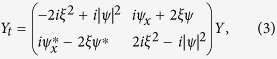


where *ξ* is a complex spectral parameter and *Y*(*t*, *x*, *ξ*) is a vector. The spatial linear operator (2) and the temporal linear operator (3) form the Lax pair of Eq. (1).

**Figure 1:**
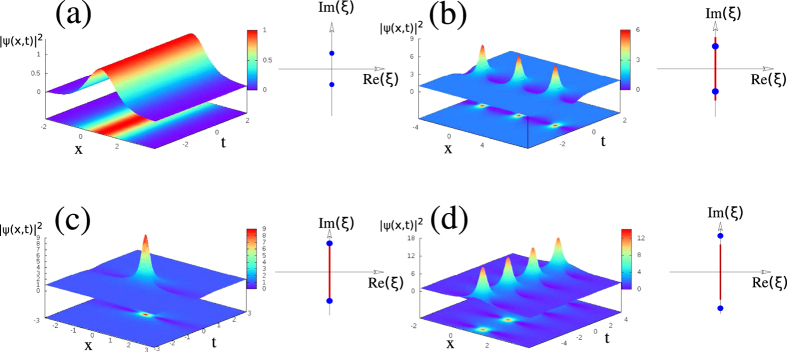
Analytical results from the IST theory. Spatio-temporal evolution (left) and spectral portraits (right) of (**a**) the fundamental soliton, (**b**) the Akhmediev breather, (**c**) the Peregrine soliton and (**d**) the Kuznetsov-Ma soliton. The red lines in spectra plotted in (**b**–**d**) represent branchcuts. The blue points in (**a**–**d**) represent complex conjugate double points.

For a given potential *ψ*(*x*, *t*) the problem of finding the spectrum {*ξ*} and the corresponding scattering solution *Y* specified by the spatial equation (2) is called the Zakharov-Shabat (ZS) scattering problem^38^. The discrete eigenvalues of the ZS operator in (2) give spectral portraits that provide precise IST signatures of various solitonic solutions of Eq. (1), which rapidly decay as 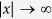. At the same time, the plane wave solution 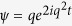 has the spectrum represented by a “branchcut” between two points *iq* and −*iq* of the simple spectrum of the *periodic* ZS problem^39^,^40^. This problem can be solved in the framework of finite-gap theory (FGT) which offers a classification of periodic and quasi-periodic solutions of Eq. (1) according to their *genus*, see Methods. The outlined spectral portraits of NLSE solitonic solutions determined from the resolution of ZS problem are shown in Fig. 1. Note that the spectrum of the periodic problem also includes the real line, which is not shown in the schematic Fig. 1 but will appear in the numerical IST spectrum plots.

As shown in Fig. 1(a), the spectrum of a stable fundamental soliton *ψ*(*x*, *t*) = *sech*(*x*)*e*^*it*^ living on a zero-background is simply made of two doubly-degenerate complex conjugate eigenvalues *ξ*_±_ = ±*i*/2. On the other hand, the spectral portraits of solitons on finite background such as ABs, PSs or KM solitons essentially represent the spectral portraits of the fundamental soliton superimposed on the spectrum of the plane wave and differing only in the relative positions of the soliton and plane wave spectra, see Fig. 1(b–d) and Methods for the mathematical description of the spectral portraits of the SFBs.

### Numerical computation of spectral portraits of soliton solutions of the 1D-NLSE

**Figure 2:**
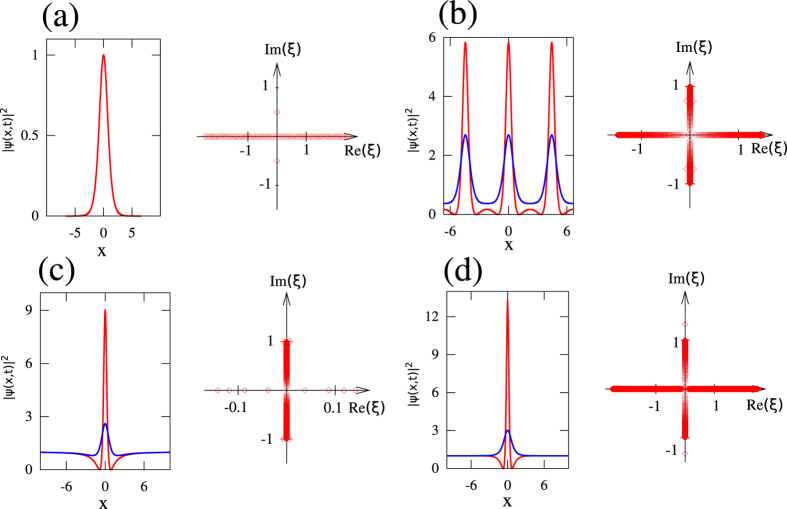
Numerical IST analysis of some soliton solutions of the 1D-NLSE. Spatial profiles (left) and spectral portraits (right) computed from numerical simulations for (**a**) the fundamental soliton 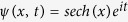, (**b**) the Akhmediev breather (Eq. (6), *ϕ* = *π*/4), (**c**) the Peregrine soliton (Eq. (6), *ϕ* = 0) and (**d**) the Kuznetsov-Ma soliton (Eq. (7), *φ* = *π*/4). The red lines represent power profiles 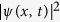 at *t* = 0 and the blue lines represent power profiles 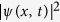 at *t* = 0.5. Red open squares represent IST spectra that are numerically computed both at *t* = 0 and at *t* = 0.5, thus showing that spectral portraits are time-independent. A numerical box of size *L* = 500 discretized by using 10000 points has been used to compute spectra plotted in (**b**–**d**).

Although the spectral portraits of soliton solutions shown in Fig. 1 are given by the IST theory^41^,^42^,^43^, the more general wave structures are often difficult to analyze, and some numerical procedures have also been developed to compute IST spectra^30^,^38^. In our numerical simulations, we have used a procedure in which Eq. (2) is rewritten as a standard linear eigenvalue problem that is subsequently solved by using the Fourier collocation method (see the Methods). Figure 2 shows the spectral portraits that are numerically computed in this way for the fundamental soliton, the AB, the KM soliton and the PS.

Some important remarks must be made regarding the use of the numerical procedure implemented to compute the IST spectra. So far, this procedure has been proven to be efficient and reliable for the computation of IST spectra of decaying potentials, such as solitons on zero background^38^. The correct numerical computation of IST spectra of decaying potentials is achieved when the size *L* of the numerical box is significantly greater than the typical size Δ*x* characterizing the decaying potential. The numerical IST procedure thus provides the complex conjugate eigenvalues *ξ*_±_ = ±*i*/2 of fundamental soliton *ψ*(*x*, *t*) = *sech*(*x*, *t*)*e*^*it*^ with a very good accuracy as far as the size *L* of the box used for numerical simulations is at least ten times greater than the typical size Δ*x* ~ 1 of the fundamental soliton, see Fig. 2(a).

To the best of our knowledge, the numerical determination of the spectral portraits of non-decaying solutions belonging to the family of ABs, KM and PS has not been made before our work. As for the fundamental soliton, the IST spectrum of SFB must be computed by taking a numerical box having a size *L* greater than the typical width Δ*x* of the SFB. However *L* must now be *much greater* than Δ*x*, to capture the important part of the spectral portrait related to the non-zero background – the branchcut, or the “spine”^44^,^45^, corresponding to the spectral band. The spectra plotted in Fig. 2(b–d) have been computed by taking boxes having a size *L* = 500 that is much greater than the typical width Δ*x* ~ 1 of the SFB under consideration. Reducing the size *L* of the numerical box while keeping the same number of points used for discretizing the SFB, the density of spectral points found inside the branch cut region of the spectrum (i.e. *ξ* ∈ [−*i*, *i*]) decreases. If the size *L* of the numerical box becomes comparable to the typical width Δ*x* of the SFB (i.e. *L* < 10Δ*x*), the branchcut can even be lost and the spectral signatures numerically determined for the SFB just become two complex conjugate eigenvalues, as for the fundamental soliton (see also Supplementary Section).

Note that the IST spectra of SFBs given by Eqs (6 and 7) do not depend on time *t* in agreement with the IST theory. This is illustrated in Fig. 2(b–d) which shows that despite the fact that 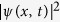 significantly changes between *t* = 0 (red line) and *t* = 0.5 (blue line), the IST spectra of the AB, KM and PS do not change in time.

### Spectral portraits of periodized structures

In this paper, we show that numerical IST analysis can be implemented to get a highly accurate spectral signature of noise-generated structures that are found in the 1D-NLSE problem with random initial conditions. However, the implementation of this numerical procedure is not quite straightforward, and we show in this Section that the correct determination of IST spectra of localized structures within more general solutions of the 1D-NLSE requires the IST analysis of *periodic* wavetrains.

As discussed above, the IST spectrum of SFBs is not qualitatively properly determined if the size *L* of the numerical box is comparable to the spatial width Δ*x* of the analyzed SFB (i.e. *L* < 10Δ*x*). In other words, the truncation procedure that consists in performing a *local* IST analysis of an isolated SFB amounts to ignoring the nonlinear interaction between the isolated part of the SFB and surrounding structures. e.g. by considering one isolated period of an AB for the numerical IST analysis in a box of the size *L* = 2*π*/*p*, we perform the analysis of an isolated object that does not interact with the neighboring oscillations within the periodic AB structure. Although this isolated object locally appears to be practically identical to an AB, the effect of the nonlinear interaction of the isolated object with the neighbors is lost. As a result, in these conditions, the numerical IST analysis does not yield the IST spectrum that is known analytically simply because the analyzed object is globally not an AB. Thus, the interaction between the modes is essential for the correct identification of an AB, and also should be taken into account in the identification of any RW object, as also shown in Supplementary Section.

**Figure 3:**
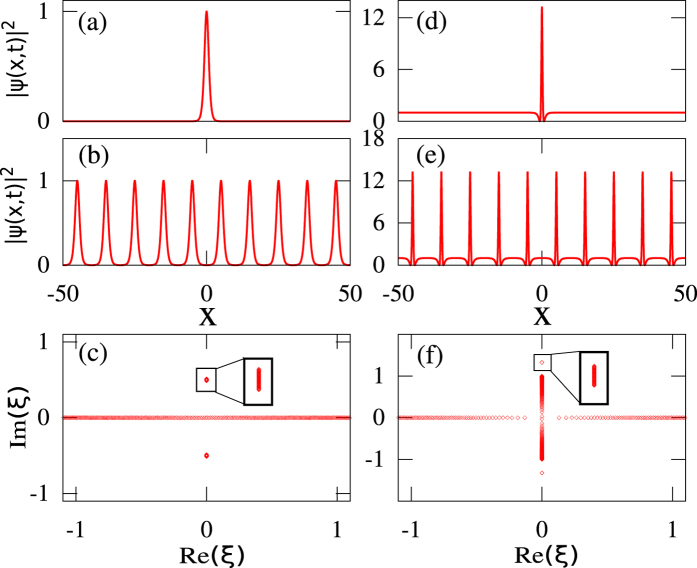
Numerical IST analysis of periodized waveforms. (**a**) Spatial profile of a fundamental soliton 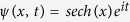. (**b**) Spatial profile of the fundamental soliton periodized in space with a period Λ = 10. (**c**) Spectral portrait of the periodized soliton showing that the periodization procedure produces a band having a small size. (**d**) Spatial profile of the KM soliton (Eq. (7), *φ* = *π*/4). (**e**) Spatial profile of the KM soliton periodized in space with a period Λ = 10. (**f**) Spectral portrait of the periodized KM soliton showing that the periodization procedure produces a band having a small size.

To overcome the fact that a satisfactory numerical IST analysis of RWs cannot be generally achieved in a local way from a single isolated object (see also Supplementary Section), we introduce here the idea that the spectral portrait can nevertheless be accurately determined from the IST analysis of an isolated object, *that has been appropriately made periodic in space*. The theoretical motivation and numerical justification of this idea are presented in the next section and in the Supplementary Section. This is illustrated in Fig. 3 that shows that the IST spectra obtained from the procedure in which localized structures are truncated to their central core part which is subsequently repeated to form a periodic function. As shown in Fig. 3(a–c) for the fundamental soliton and in Fig. 3(d–f) for the KM soliton (see Supplementary Section for the results related to the PS), the IST analysis of periodic trains that are produced in this way provides the IST spectra that are very close to the spectra of the pure and non-periodic objects, see Fig. 2(a,c). The major difference between the IST spectra plotted in Fig. 2(a,c) and the IST spectra plotted in Fig. 3(c,f) lies in the fact that small bands are now found instead of single points.

By producing a periodic extension of an isolated localized object, we realize a local finite-band approximation of the wave field and thus, no longer ignore the nonlinear interactions between the object and the surrounding structure. The spatial period Λ that is used to produce the periodic waveform defines the effective intensity of the interactions, which is translated into the width of the bands in the IST spectrum whereas the detailed shape of the extracted object determines the number and location of the bands. The larger the period Λ is, the smaller the bands found in the IST spectrum are. Therefore the choice of the spatial period Λ is crucial for the quantitatively correct determination of the local IST spectrum in our numerical procedure. That being said, for spatially isolated structures such as the genuine fundamental soliton, PS or KM soliton, the choice of the period of the numerical IST is not essential as long as the period is much greater than the typical soliton width. Indeed, our numerically computed spectra for the fundamental soliton and KM soliton are very similar to the exact IST spectra of the respective exact solutions shown in Fig. 2 as long as Λ is chosen in such a way that the pattern isolated before periodization includes the soliton part of the SFB together with some part of the background (see e.g. Fig. 3(f)). The criteria for the choice of Λ providing a robust spectral portrait will be discussed in the next Section.

The described procedure has some instructive parallels with the analysis of dispersive shock waves (DSWs)^46^: in some cases DSWs can be viewed as purely solitonic wave trains^47^, although generally, they are more accurately represented by the modulated periodic (genus one) solutions of the relevant equation and exhibit near-solitonic properties only in the vicinity of one of the edges. Thus the local IST spectrum a DSW can be captured only by considering a periodic wavetrain, not a localized pulse, the period being defined by the distance between the neighboring oscillations.

### Dam break problem and the generation of rogue waves

In this Section, we use the tool of numerical IST analysis of periodized waveforms to investigate the generation of RWs in the context of the dam break problem recently considered in ref. 48. The dam break problem represents an analytically tractable scenario of the RW formation in the framework of the focusing NLSE (1). The evolution of an initial condition having the shape of a rectangular barrier considered in the small dispersion limit of Eq. (1) enables the generation of the periodic or quasi-periodic nonlinear wave structures containing many oscillations which can be described within the semi-classical approximation. During the initial stage of the evolution these structures are described by the modulated single-phase (genus one) NLSE solutions and can be associated with DSWs. With the barrier initial condition, the interaction between two counter-propagating DSWs has been shown in^48^ to lead to the emergence of a modulated two-phase large-amplitude breather lattice whose amplitude profile can be approximated by ABs or PS within certain space-time regions. More generally, it was shown that the structures closely resembling ABs and PSs actually represent modulated two-phase (genus 2) NLS solutions.

**Figure 4:**
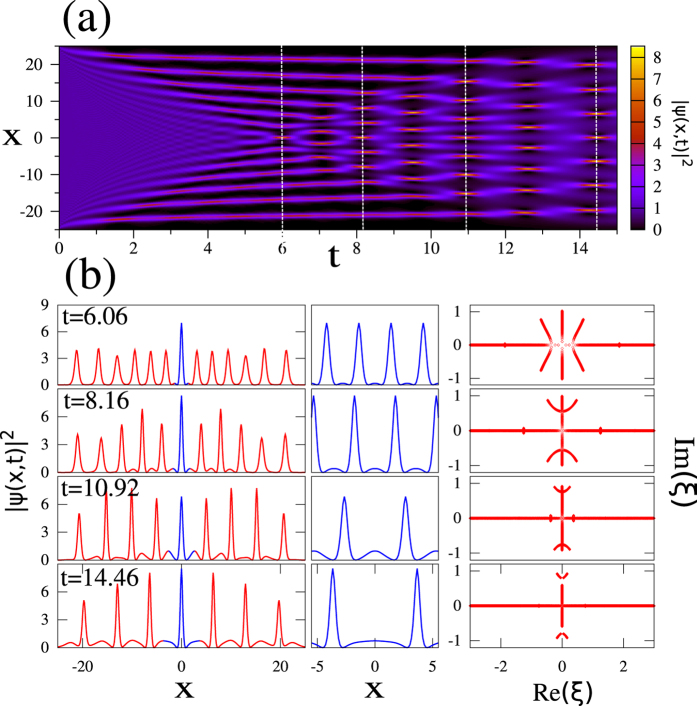
Dam break problem. (**a**) Space-time diagram showing the evolution of the power 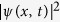 of the wave while starting from the “box” initial condition given by Eq. (4) (*l* = 25). Eq. (1) is integrated by using a numerical box having a size *L* = 512. (**b**) Numerical IST analysis of periodized waveforms. Profiles of the power 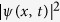 at times *t* = 6.06, *t* = 8.16, *t* = 10.92, *t* = 14.46 are plotted in red in the left column. The parts of the profiles that are highlighted in blue around *x* = 0 represent the elementary patterns that are periodized to produce waveforms shown in the central column. The spectral portraits plotted in the right column are computed from the numerical IST analysis of the periodic waveforms shown in the central column. The numerical IST analysis is made from periodic waveforms including 500 periods.

The spatio-temporal diagram plotted in Fig. 4(a) shows the time evolution of the power 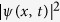 while starting from an initial condition given by:


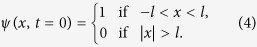


Note that we solve numerically a problem with zero boundary conditions (i.e. the size *L* = 512 of the box used for numerical simulations of Eq. (1) is bigger than the size 2*l* = 50 of the rectangular barrier). As shown in Fig. 4(a) and extensively discussed in ref. 48, the DSW collision leads to the formation of high-power narrow structures localized around *x* = 0. These localized structures observed at *t* = 6.06, *t* = 8.16, *t* = 10.92, *t* = 14.46 are highlighted in blue in the left column of Fig. 4(b). The spatial size Λ of localized structures that are analyzed by our numerical IST procedure is defined by the distance separating the maxima reached by the two side lobes surrounding the localized peak of interest, see blue lines in the left column of Fig. 4(b). The isolated patterns highlighted in blue are periodized (see central column in Fig. 4(b)) and the numerical IST analysis is then made from periodic waveforms including 500 periods. Any sufficiently small change in the spatial size Λ will produce IST spectra that are quantitatively slightly different from those plotted in the right column of Fig. 4(b). However, as our simulations have shown, the general result of numerical IST analysis is robust and will not be qualitatively changed as far as the elementary pattern includes the peak centered around *x* = 0 together with some parts of the side lobes. The size Λ of the elementary pattern characterizes the effective interaction domain of the central peak with the surrounding structure. Generally one can propose the following criterion for the correct choice of Λ: the period Λ is chosen correctly if any sufficiently small change in Λ produces only small quantitative change in the spectrum. Some structures (like exact ABs) could be more sensitive to the variations of Λ than others.

The right column of Fig. 4(b) shows the spectral portraits of “rogue-like” peaks emerging from the dam break scenario. All the IST spectra reveal the presence of 3 main spectral bands, thus confirming that the observed structures represents genus 2 solutions of the 1D-NLSE^48^. The localized structures observed around *x* = 0 at *t* = 8.16 and at *t* = 14.46 are very close to the PS in the sense that they can be locally very well fitted by a profile given by Eq. (6). However the numerical IST analysis made at *t* = 8.16 and *t* = 14.46 reveals that those localized structures represent non-degenerate genus 2 solutions of Eq. (1) that are not identical to the PS (compare IST spectrum of Fig. 2(c) with IST spectrum of Fig. 4(b)). All the above features fully agree with the analytical results of ref. 48 supporting the effectiveness of our numerical approach.

It should be stressed that the rectangular barrier problem can be, in principle, solved using the classical IST method with zero boundary conditions. In the problem with a wide initial barrier (or, equivalently, with small dispersion parameter — see ref. 48) the exact, global, IST spectrum has both discrete and continuous component with a large number of discrete eigenvalues concentrated along the imaginary axis and *remaining constant in time*. However, while this spectrum implies a long-time asymptotic outcome dominated by a large number of fundamental solitons, it says little about the nonlinear wave field at intermediate times. The appearance of the finite-band dynamics, locally approximating the exact solution’s behavior at intermediate times, is the result of a complex nonlinear interaction between the “elementary IST modes”. The genus of the “effective” finite-band potential, appearing as a result of this interaction, as well as the location and size of the spectral bands, are the definitive parameters, which, in particular, characterize proximity of the observed RW structures to the classical SFBs.

### Noise-driven modulational instability and the generation of rogue waves

In this Section, we use the tool of the numerical IST analysis of periodized waveforms to determine the nature of localized structures that are found in the context of the so-called noise-driven modulational instability (MI)^6^,^20^,^23^. The theoretical description of the nonlinear stage of MI is now a challenging question of fundamental importance^23^,^49^. Extensive numerical simulations have shown that coherent structures localized in space and time may emerge from noise through the process of MI that is initiated by a random perturbation of an initial plane wave^6^,^8^,^9^,^20^,^23^. The question of the identification and of the characterization of these localized structures has recently received special attention in the context of RW generation. Using fitting procedures, it has been shown that some of these localized structures can be *locally* well approximated by analytic SFB solutions given by Eq. (6) or by Eq. (7) 6,8,9,20,21.

We implement here the numerical IST analysis to get accurate spectral signatures of some typical noise-generated structures that are found in the 1D-NLSE problem with random initial conditions. Our study shows that those localized structures correspond to a variety of *non-degenerate* genus 2 and genus 4 solutions of Eq. (1) that differ from the *degenerate* genus 2 solutions given by Eq. (6) or by Eq. (7). The proposed numerical IST procedure thus provides a new insight into the characterization of the RWs found in random wave trains.

**Figure 5:**
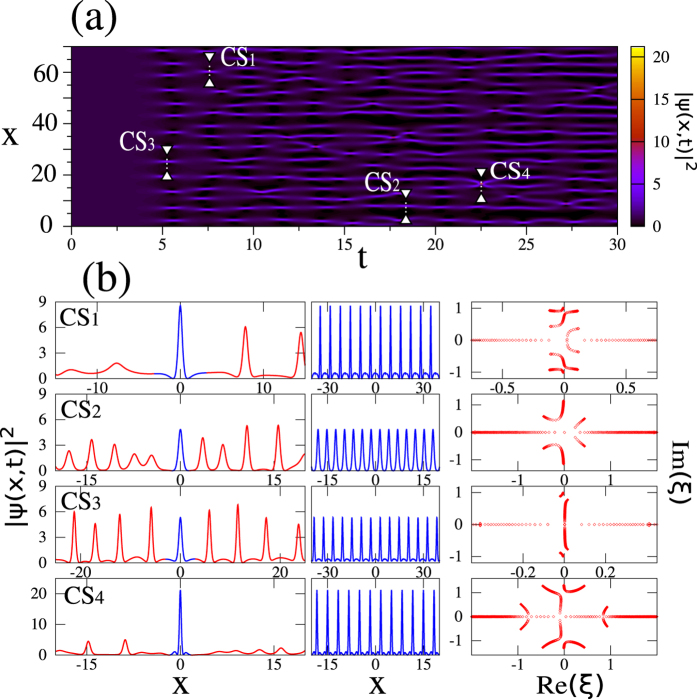
Noise driven modulational instability. (**a**) Space-time diagram showing the evolution of the power 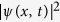 of the wave while starting from the initial condition given by Eq. (5). (**b**) Coherent structures are extracted from random profiles fluctuating in space *x* in specific regions labeled *CS*_1_, *CS*_2_, *CS*_3_ in (**a**). The profiles highlighted in blue in the left column represent the basic patterns that are periodized to produce waveforms shown in the central column. The spectral portraits plotted in the right column are computed from numerical IST analysis of periodic signals shown in the central column and including 200 periods. The region labeled *CS*_4_ in (**a**) is a region in which a strongly localized and intense peak is observed. The bottom row of (**b**) shows the IST spectrum (right column) computed from the periodization of this big peak.

The spatio-temporal diagram plotted in Fig. 5(a) shows the time evolution of the power 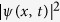 while starting from an initial condition given by:


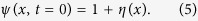


*η*(*x*) is a small complex noise field computed from the inverse Fourier transform of a broadband spectrum under the assumption of a random phase process, see Methods. As shown in Fig. 5(a), the spatio-temporal evolution found from our numerical simulations of Eq. (1) is qualitatively very similar to the one evidenced in refs 6 and 20.

Figure 5(b) shows the results obtained from the IST analysis of coherent structures which are found in the regions labeled *CS*_1_, *CS*_2_, *CS*_3_, *CS*_4_ in Fig. 5(a). The first row of Fig. 5(b) shows that the coherent structure *CS*_1_ having a peak power close to 9 is a genus 4 solution of the 1D-NLSE. The analysis of the periodized signal (central column in Fig. 5(b)) indeed reveals a IST spectrum including 5 main bands (right column in Fig. 5(b)). On the other hand, coherent structures *CS*_2_ and *CS*_3_ shown in the second and third rows of Fig. 5(b) have a IST spectrum made with 3 bands. The coherent structures *CS*_2_ and *CS*_3_ extracted from wavetrains nearly periodic in time and in space (see Fig. 5(a)) are therefore genus 2 solutions of Eq. (1). Although the IST spectrum of *CS*_3_ is concentrated around the vertical imaginary axis, it is however relatively far from the IST spectra of SFBs given by Eq. (6) or Eq. (7), see Fig. 2(b–d) and also Supplementary Section for a discussion about results of best fit approximations of *CS*_1_ − *CS*_4_.

The region labeled *CS*_4_ in Fig. 5(a) is a region where a collision occurs between two SFBs in the (*x*, *t*) plane. A large peak with a maximum intensity of ~22 is formed as a result of this collision. Such a localized and intense event has already been observed in numerical simulations reported in refs 8 and 20 where it has been fitted with a rational breather of order 2, a degenerate genus 4 solution of Eq. (1). The IST spectrum of the second-order rational breather is made of 5 spectral bands that have collapsed to form the IST spectrum consisting of a branch cut and two complex conjugate points each having quadruple degeneracy. The fourth row Fig. 5(b) shows that the coherent structure *CS*_4_ resulting from the collision between two SFB is a genus 4 solution of Eq. (1) because its IST spectrum is composed of 5 main bands. However, *CS*_4_ is not a degenerate genus 4 solution of Eq. (1) and the analysis of its IST spectrum allows one to clearly distinguish this high-amplitude coherent structure from the exact second-order breather solution considered in refs 8 and 20 (see also Supplementary Section for results about the best fit of *CS*_4_).

## Discussion and Conclusion

From the theoretical point of view, the 1D-NLSE with periodic boundary conditions and random initial conditions can be solved analytically in the framework of the FGT because any periodic solution of Eq. 1 can be approximated by a finite-band potential expressed in terms of Riemann theta functions over certain algebraic curve^23^,^45^. However, this general mathematical result is very difficult to implement in practice because the genus of the solution is too large for initial conditions involving a large number of Fourier modes with random phases. The numerical realization of the global FGT analysis thus requires very significant computing resources^45^. In contrast, we perform a local finite-band approximation of the wave field which includes only the most essential nonlinear interactions. Our approach thus provides the effective IST spectra giving an accurate signature of the nature of the isolated pulse and brings a new insight into the problem of the characterization of RWs and the mechanisms leading to their formation in integrable turbulence^21^,^23^.

There has been an extensive work on the emergence of the specific SFBs described by Eqs (6) and (7), and their higher-order collisons^8^,^20^. However, the randomness and the interactions among the structures are the factors that prevent the emergence of *exact* SFBs in integrable turbulence. The IST spectra enable one to quantify the differences between specific exact solutions of 1D-NLSE and the observed localized structures. Indeed, the localized structures found in the spatio-temporal evolution plotted in Fig. 5(a) correspond to a variety of *non-degenerate* genus 2 and genus 4 solutions of Eq. (1) that differ from the particular *degenerate* genus 2 solutions given by Eq. (6) or by Eq. (7) or the degenerate genus 4 solutions corresponding to higher-order rational breathers^8^. Although we have not performed an extensive statistical analysis of the content of random wave trains, it is very unlikely that exact *degenerate* genus 2 solutions given by Eq. (6) or by Eq. (7) can be found in the noise-driven evolution of the focusing 1D-NLSE.

The understanding of the statistics of RWs in integrable turbulence is an open and complex question^21^,^23^. In particular, it has been shown that the stationary probability density function (PDF) of the field is gaussian if the initial stage consist of a condensate with additional noise^23^ whereas the stationary PDF is strongly non gaussian if the initial stage is a partially coherent wave^21^. Islas and Schober^36^ have proposed to correlate the occurrence of RWs with the proximity to homoclinic solutions of the 1D-NLSE. The degree of proximity to homoclinic solutions is determined in ref. 36 by some quantitative measurements over the IST spectrum. In a very recent paper^27^, another approach has been used and the “global” IST spectra of random initial conditions have been computed to study the appearance of RWs during the NLS evolution. We stress that, in contrast to the above two works, the aim of the analysis of our paper is not to predict the RW occurrence but rather to perform an accurate local characterization of coherent structures already existing in a globally incoherent nonlinear wave field. As shown in ref. 50, the maximum amplitude of a finite-gap solution to the focusing 1D-NLSE with given spectral bands does not exceed half of the sum of the length of all the bands. Using this criterion, the IST spectrum of a local periodized coherent structure can be used to measure the maximum amplitude possibly reached by this coherent structure. Moreover, our approach could be combined in the future with IST based predictive methods to obtain a statistical treatment of NLSE rogue waves.

## Methods

### Mathematical expressions describing the AB, PS and KM soliton and their spectral portraits

ABs correspond to solutions of Eq. (1) that are periodic in space but localized in time. The AB solution of Eq. (1) can be expressed in terms of a single real parameter *ϕ*:


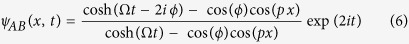


where 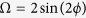 and 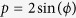. Figure 1(b) shows the IST portrait of an AB. The complex conjugate double points are given by 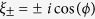 and the endpoints of the branchcut are 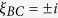. The family of KM solutions corresponds to SFB that are periodic in time *t* and localized in space *x*. It can be also expressed in terms of a single real parameter *φ*:


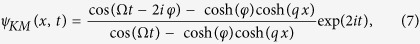


where 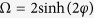 and 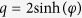. Figure 1(d) shows the IST portrait of a KM soliton. The complex conjugate double points are given by 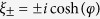 and the endpoints of the branchcut are 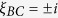, as for the AB. In the limit where *ϕ* → 0 or *φ* → 0, the period of AB and KM solutions tends to infinity and the solution of Eq. (1) that is localized both in space and time is named Peregrine soliton. In the spectral portrait of the PS, the complex conjugate double points coincide with the endpoints of the branchcut 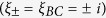, as shown in Fig. 1(c).

### Numerical Simulations

The determination of discrete eigenvalues *ξ* of the Zakharov-Shabat system is made by rewriting Eq. (2) as a standard linear eigenvalue problem


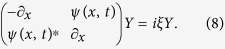


The *x*–axis is truncated into a finite box of size *L*. The eigenvector 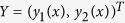 as well as the potential 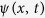 are expanded into Fourier series with 2*n* + 1 modes. These Fourier expansions are substituted in Eq. (8) and the obtained system for the eigenvalues is then solved by using standard linear algebra routines^38^. IST spectra plotted in Fig. 2 have been obtained by taking boxes of size *L* = 500 that have been discretized by using 10^4^ points. IST spectra plotted in Fig. 3 and in Fig. 4(b) have been obtained from series including 500 periods that have been discretized by using more than 2.10^4^ points. IST spectra plotted in Fig. 5(b) have been obtained from series including 200 periods that have been discretized by using more than 2.10^4^ points.

Numerical simulations of Eq. (1) have been performed by using a pseudo-spectral method working with a step-adaptative algorithm permitting one to reach a specified level of numerical accuracy. In Fig. 4, a numerical box of size *L* = 512 has been discretized by using 2^14^ points. In Fig. 5, a box of size *L* = 2000 has been discretized by using 2^16^ points.

The random complex field *η*(*x*) used as initial condition in Eq. (5) is made from a discrete sum of Fourier components:


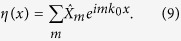


with 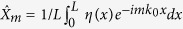 and *k*_0_ = 2*π*/*L*. The Fourier modes 
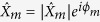
 are complex variables. We have used the so-called random phase (RP) model in which only the phases *ϕ*_*m*_ of the Fourier modes are considered as being random^51^. In this model, the phase of each Fourier mode is randomly and uniformly distributed between −*π* and *π*. Moreover, the phases of separate Fourier modes are not correlated so that 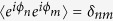 where *δ*_*nm*_ is the Kronecker symbol (*δ*_*nm*_ = 0 if *n* ≠ *m* and *δ*_*nm*_ = 1 if *n* = *m*). With the assumptions of the RP model above described, the statistics of the initial field is homogeneous, which means that all statistical moments of the complex field *η*(*x*) do not depend on *x*^52^. In the RP model, the power spectrum *n*_0_(*k*) of the random field *η*(*x*) reads as:


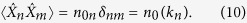


with *k*_*n*_ = *nk*_0_. In our simulations, we have taken a random complex field *η*(*x*) having a gaussian optical power spectrum that reads


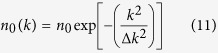


where Δ*k* is the half width at 1/*e* of the power spectrum. The values of *n*_0_ and Δ*k* taken in our numerical simulations are *n*_0_ = 5.645.10^−3^ and Δ*k* = 0.5.

### Finite-Gap Theory

The periodic ZS problem is generally solved in the class of the so-called finite-band potentials which are non-decaying periodic or quasi-periodic NLSE solutions having the ZS spectrum filling several bands of finite width^40^,^44^,^45^. The finite-band (or as it is often called, finite-gap) theory (FGT) is widely recognized as a natural framework for the analysis of nonlinear modulational instability and the formation of RWs although its practical implementation for the analytic description of integrable turbulence encounters some fundamental difficulties^23^. Within the FGT the multi-phase NLSE solutions are characterized by a *genus*, calculated as *N* − 1, where *N* is the number of spectral bands. Physically, the genus characterizes the number of degrees of freedom (i.e. the number of fundamental oscillatory modes, or phases) within the nonlinear periodic or quasiperiodic solution for the envelope of the plane wave^45^. Mathematically, the solution genus represents the genus of the hyperelliptic Riemann surface, on which the finite-band NLSE solution is defined in terms of theta-functions^45^. From the viewpoint of the FGT, the plane wave itself is classified as a regular genus 0 solution while the fundamental soliton represents a degenerate genus 1 solution with two complex conjugate, doubly-degenerate spectral points of the periodic problem, the counterparts of the discrete spectrum in the ZS problem with decaying potentials. The standard SFBs (ABs, KM solitons and PSs) all are the degenerate genus 2 solutions. Their spectral portraits determined from the resolution of ZS problem are shown in Fig. 1.

## Additional Information

**How to cite this article**: Randoux, S. *et al*. Inverse scattering transform analysis of rogue waves using local periodization procedure. *Sci. Rep.* **6**, 29238; doi: 10.1038/srep29238 (2016).

## Supplementary Material

Supplementary Information
