# Supplementary material for: Inverse scattering transform analysis of rogue waves using local periodization procedure
Source: Sci Rep. 2016 Jul 7;6:29238. doi: 10.1038/srep29238 (PMC4935857; doi:10.1038/srep29238)
Supplement: Supplementary Information [file srep29238-s1.pdf]

Supplementary Information: Inverse scattering transform analysis of rogue waves using local periodization procedure

Stéphane Randoux,[1] Pierre Suret,[1] and Gennady El[2]

[1]*Laboratoire de Physique des Lasers, Atomes et Molecules, UMR-CNRS 8523, Université de Lille, France*
[2]*Department of Mathematical Sciences, Loughborough University, Loughborough LE11 3TU, United Kingdom*

I. THE ROLE OF THE PERIODIZATION PROCEDURE IN CAPTURING CORRECT SPECTRAL PORTRAITS

In this Section, we use the example of the dam-break problem to show that the “local periodized” numerical IST analysis enables one to capture the asymmetry of the interaction of a local coherent structure with the surrounding wave field. Moreover, we demonstrate that the procedure of periodization is essential for getting the correct spectral signature of the local coherent structures under consideration.

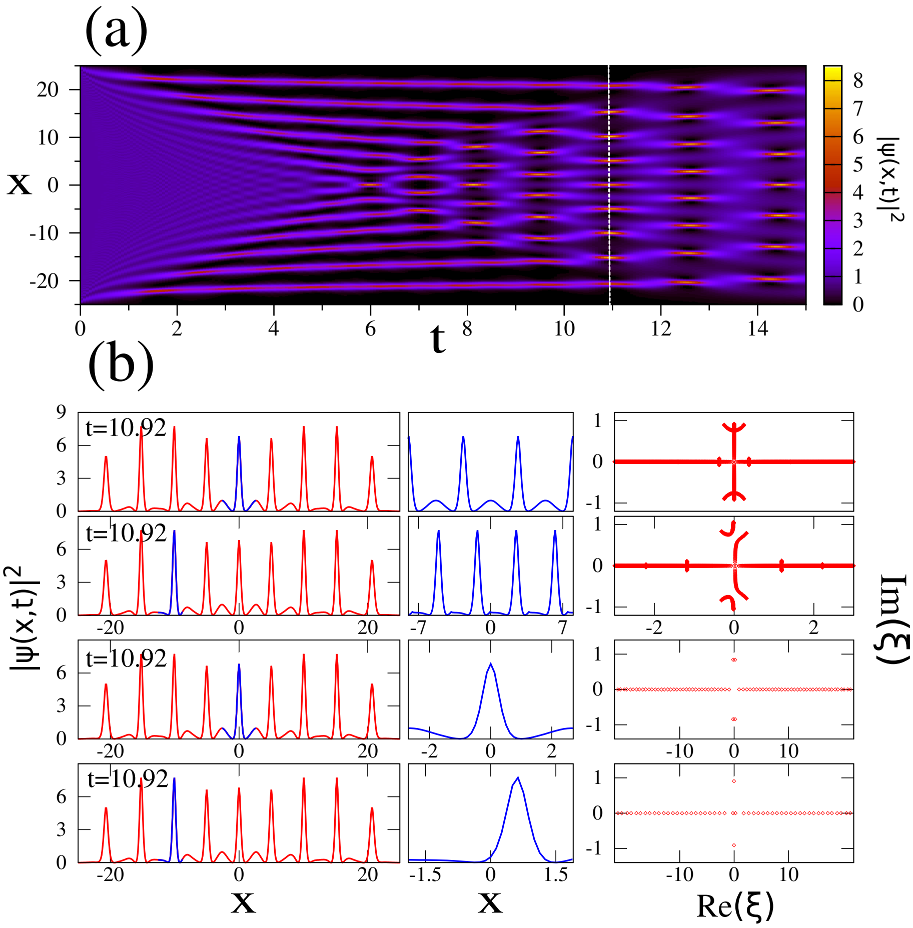

Figure S1: **Dam break problem**. (a) Space-time diagram showing the evolution of the power $|\psi(x,t)|^2$ of the wave while starting from the “box” initial condition given by Eq. (4) of our manuscript. (b) Numerical IST analysis of some waveforms. The power profile $|\psi(x,t)|^2$ at time $t = 10.92$ is plotted in red in the left column. **In the two top rows**, the parts of the profiles that are highlighted in blue around $x = 0$ and $x = -10$ represent the elementary patterns that are periodized to produce waveforms shown in the central column. The spectral portraits plotted in the right column are computed from the numerical IST analysis of the periodic waveforms shown in the central column (numerical IST analysis made over 500 periods). **In the two bottom rows**, the parts of the profiles highlighted in blue around $x = 0$ and $x = -10$ represent the elementary patterns that are directly analyzed by IST **without using the periodization procedure.**

Fig. S1(a) shows the spatio-temporal evolution of the power $|\psi(x,t)|^2$ corresponding to the dam-break problem considered in the article. The first row in Fig. S1(b) is identical to the third row of Fig. 4(b) of the article: it represents the result of the IST analysis of the peak localized around $x = 0$ at $t = 10.92$ and shows that the local IST spectrum exhibits a symmetric genus two signature (since it is made of 3 symmetric main bands). The second row

of Fig. S1(b) represents the result of the IST analysis of the peak localized around $x = -10$ ($t = 10.92$). The IST analysis of the periodized waveform reveals that this coherent structure is approximated by a genus 2 solution of the 1D-NLSE too. However, in contrast to the peak localized around $x = 0$, the IST spectrum has lost the symmetry with respect to the imaginary axis ($Re(\xi) = 0$) because the analyzed structure is no longer spatially symmetric. This asymmetry of the spectrum reflects the asymmetry of the interaction of the chosen local coherent structure with the surrounding wave field and is acutely captured by our periodized procedure.

Summarizing, the "local periodized" numerical IST analysis of the dam break (barrier) problem shows that the coherent structures emerging at intermediate times within certain x, t-region can be classified as genus 2 solutions of the 1D-NLSE. The numerically found local IST spectrum is in full agreement with the theoretical results of Ref. [1].

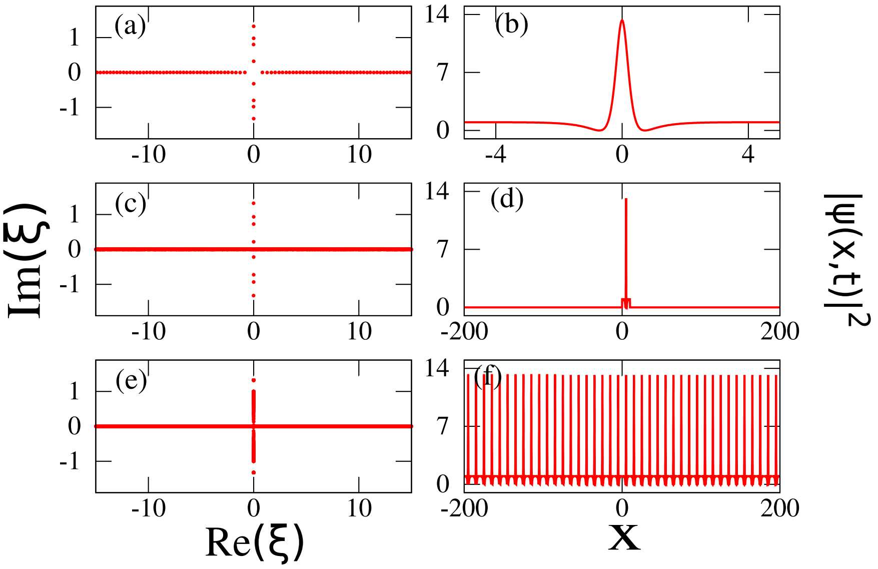

Figure S2: Numerical IST analysis of a KM soliton (Eq. (7) of our manuscript, $\phi = \pi/4$) by using different methods. (a) (b) IST analysis of the KM soliton in a small box having a size $l = 10$. (c), (d) IST analysis of the KM soliton in a large box having a size $L = 400$. The KM soliton is truncated to its central core part in a small box having a size $l = 10$. $\psi(x)$ is set to zero outside this small box, as shown in (d). (e), (f) IST analysis of the KM soliton that has been periodized (spatial period $\Lambda = 10$), as in Fig. 3 of our manuscript.

As explained in the article, performing a IST analysis of an isolated localized structure **without applying the periodization procedure** amounts to ignoring the nonlinear interaction between this isolated structure and the surrounding wave field. If some coherent peaks are isolated at different spatial positions X (but at a given time in the dam break problem) and if their IST analysis is numerically made without using the periodization procedure, we typically obtain spectra that include two complex conjugate eigenvalues, as illustrated in the two bottom rows of Fig. S1. Thus, the IST spectrum of a coherent structure appearing locally similar to some SFB is essentially (up to some amount of the continuous component) identical to the IST spectrum of a fundamental soliton living on a zero background when the IST analysis is made without using the periodization procedure introduced in our paper. This means that, by truncating an isolated peak and making the IST analysis in a box having the size l comparable to the size of this peak, the effect of the nonlinear interaction of the isolated object with the neighbors is lost. As a result, in these conditions, the numerical IST analysis does not yield the correct IST spectrum.

This important conclusion is supported by the IST analysis of standard SFBs. Figures S2 and S3 show the results of the numerical IST analysis of the KM soliton and PS respectively, using different procedures (truncation vs periodization) for the structure under study. Without the periodization procedure, the IST analysis of an isolated KM soliton (resp. PS) made in a box having a size $l = 10$ comparable to the typical spatial size (~ 1) of the KM soliton (resp. PS) provides only a few discrete imaginary eigenvalues without capturing the correct spectrum, see Fig. S2(a)(b) (resp. Fig. S3(a)(b)). Still not applying the periodization procedure and performing the IST analysis of a PS or of a KM soliton that have been truncated to their central core part to be subsequently placed in a box having a size L much bigger than the size l of the central core part, only a few discrete eigenvalues are again obtained but the correct specrum is not captured by this procedure, see Fig. S2(c)(d) and Fig. S3(c)(d). On the other hand, Fig. S2(e)(f) demonstrates the efficiency of the periodization method for the correct determination of the IST spectrum of the KM soliton. Figs. S3(e)(f) show that the periodization procedure is similarly essential for capturing the correct spectral signature of the PS.

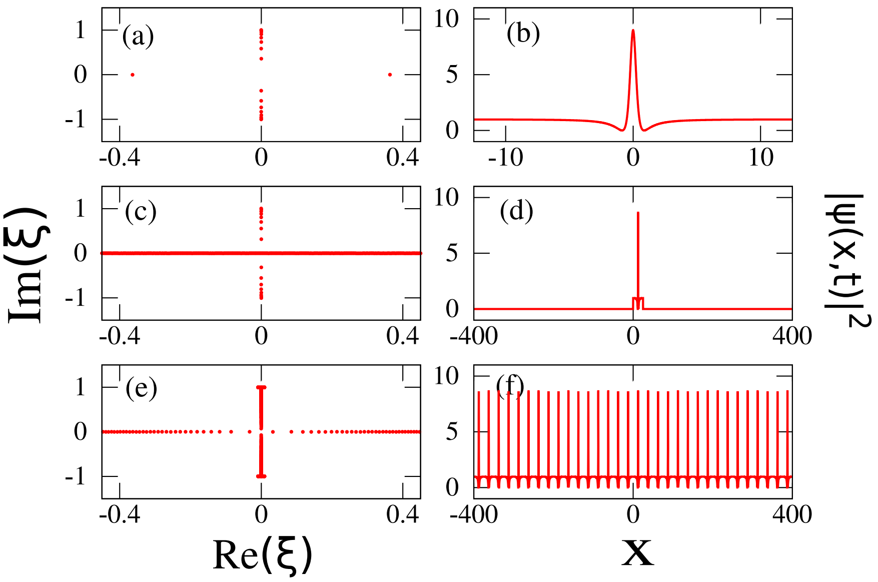

Figure S3: Numerical IST analysis of a Peregrine soliton (PS) (Eq. (6) of the manuscript, $\phi = 0$) by using different methods. (a) (b) IST analysis of the PS in a small box having a size $l = 25$. (c), (d) IST analysis of the PS in a large box having a size $L = 800$. The PS is truncated to its central core part in a small box having a size $l = 25$. $\psi(x)$ is set to zero outside this small box, as shown in (d). (e), (f) IST analysis of the PS that has been periodized (spatial period $\Lambda = 25$).

II. USE OF CONVENTIONAL FITTING PROCEDURES FOR APPROXIMATING COHERENT STRUCTURES FOUND IN THE PROBLEM OF NOISE-DRIVEN MODULATIONAL INSTABILITY

In the problem of noise-driven modulational instability, the standard procedure used for the identification of coherent structures is based on the best fit methods [2–5]. Simple analytical expressions describing the PS, the AB or the KM soliton (Eq. (6) or (7)) are usually used, and the parameters defining these expressions are adjusted to get the best possible matching between the exact SFB solution and the coherent structure that is analyzed. With this approach, strong assumptions must be made about the nature of the function that is chosen for fitting the coherent stucture under investigation.

Fig. S4(a) shows that the power profile $|\psi(x,t)|^2$ of the coherent structure CS_1 found in Fig. 5 can be well fitted by the analytical expression associated to the power profile of the PS (Eq. (6), $\phi = 0$, $t = 0$). As shown in Fig. S4(b), the phase profile $\Phi(x,t) = Arg(\psi(x,t))$ of CS_1 (red line) is also found to be in reasonable quantitative agreement with the phase profile (blue line) of the exact PS ($\psi(x,t) = |\psi(x,t)| \exp(i\Phi(x,t))$). Despite differences of relatively small importance between the profiles found in numerical simulations and the theoretical profiles characterizing the PS, the IST spectrum of CS_1 is found to exhibit significant qualitative and quantitative differences with the IST spectrum of the PS (compare IST spectrum of CS_1 in Fig. 5(b) with IST spectrum of the PS in Fig. 2(c)). In particular the local IST analysis demonstrates that CS_1 cannot be identified as a genus 2 solution of the 1D-NLSE but as a genus 4 solution. This demonstrates that local IST analysis is a very sensitive tool that is able to capture accurate signatures of coherent structures found in random wave trains.

Waveforms plotted in red lines in Fig. S4(c) and in Fig. S4(d) represent the power and the phase profiles of the coherent structure CS_3 found in Fig. 5. These profiles can be very well fitted by the analytical expression characterizing the AB (Eq. (6), $\phi = \pi/6$, $t = 0.21$). The differences between the fitted profiles and the actual profiles are very small in the specific case of CS_3. Although the IST local analysis reveals that CS_3 is a genus 2 solution of the 1D-NLSE (see Fig. 5(b)), the IST spectrum of CS_3 does not, however, coincide with the IST spectrum of an AB, see Fig. 2(b).

The power profile of the coherent structure CS_4 can be very well fitted by using a second-order rational solution of the 1D-NLSE (Eq. (4) of ref. [5], $t = -0.062$), see Fig. S4(e). The phase profile of CS_4 (red line) coincides over a wide spatial range with the phase profile of the second-order rational solution of the 1D-NLSE (blue line), see Fig. S4(f). However the existing differences between the actual and the fitted profiles explain why the IST spectrum of CS_4 is far from being degenerate (i.e. the 5 bands of the IST spectrum of CS_4 do not merge along the imaginary vertical axis, see Fig. 5(b)). Therefore, CS_4 should be identified as a general genus 4 solution of Eq. (1) that differs from the very specific degenerate analytical solution found in ref. [5].

Note that we have not been able to fit in a correct way the coherent structure CS_2 found in Fig. 5 by simple analytical expressions describing SFBs or solitons living on zero background. This is consistent with the fact that the

IST spectrum of CS_2 is "far" from IST spectra of SFBs or solitons on zero background, see Fig. 5(b) and Fig. 2.

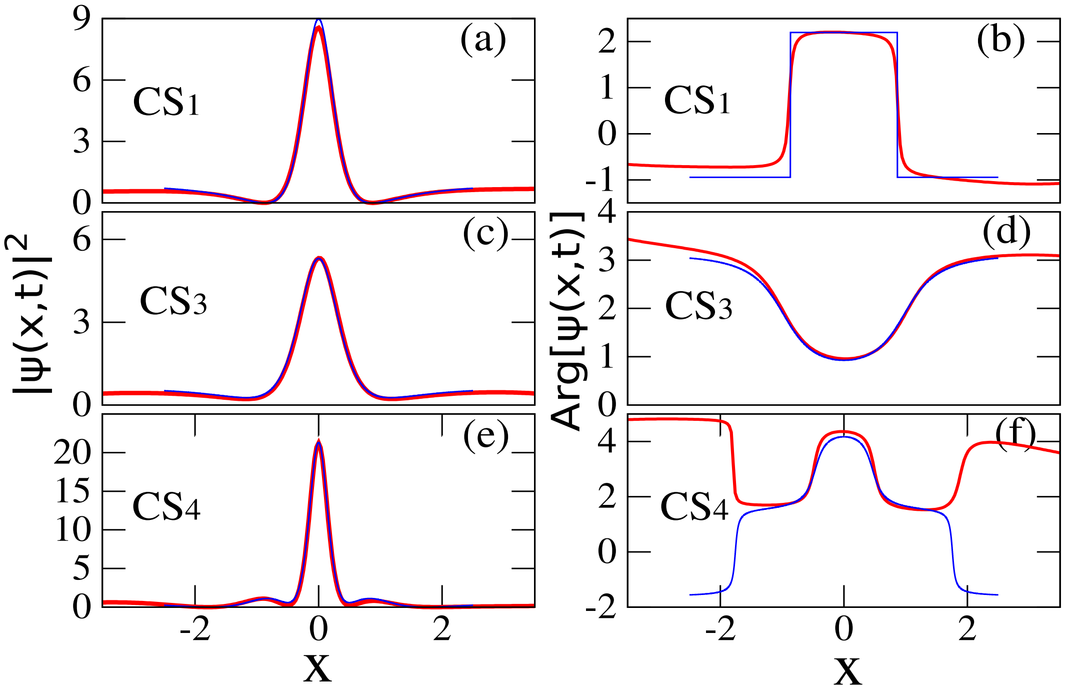

Figure S4: Left column: power profiles (red lines) $|\psi(x,t)|^2$ of three of the coherent structures (CS_1, CS_3, CS_4) isolated in Fig. 5. Right column: phase profiles (red lines) $\Phi(x,t) = Arg(\psi(x,t))$ of three of the coherent structures (CS_1, CS_3, CS_4) isolated in Fig. 5. In (a), (b), the coherent structure CS_1 is fitted (blue lines) by using the function describing a PS (Eq. (6), $\phi = 0$, $t = 0$). In (c), (d), the coherent structure CS_3 is fitted (blue lines) by using the function describing an AB (Eq. (6), $\phi = \pi/6$, $t = 0.21$). In (e), (f) the power profile of CS_4 is fitted (blue lines) by using a function describing a second-order rational solution of the 1D-NLSE (Eq. (4) of ref. [5], $t = -0.062$)

[1] El, G. A., Khamis, E. G. & Tovbis, A. Dam break problem for the focusing nonlinear schrödinger equation and the generation of rogue waves. *arXiv:1505.01785v2* (2015).

[2] Dudley, J. M., Dias, F., Erkintalo, M. & Genty, G. Instabilities, breathers and rogue waves in optics. *Nat. Photon.* **8**, 755 (2014).

[3] Akhmediev, N., Ankiewicz, A. & Soto-Crespo, J. M. Rogue waves and rational solutions of the nonlinear schrödinger equation. *Phys. Rev. E* **80**, 026601 (2009).

[4] Toenger, S. *et al.* Emergent rogue wave structures and statistics in spontaneous modulation instability. *Scientific Reports* **5** (2015).

[5] Akhmediev, N., Soto-Crespo, J. & Ankiewicz, A. Extreme waves that appear from nowhere: On the nature of rogue waves. *Physics Letters A* **373**, 2137 – 2145 (2009).
